# Supplementary material for: Universal optimal geometry of minimal phoretic pumps
Source: Sci Rep. 2019 Jul 25;9:10788. doi: 10.1038/s41598-019-46953-8 (PMC6658517; doi:10.1038/s41598-019-46953-8)
Supplement: Supplementary file 1 — Supplementary Material for Universal optimal geometry of minimal phoretic pumps [file 41598_2019_46953_MOESM1_ESM.pdf]

Supplemental Material for:
"Universal optimal geometry of minimal phoretic pumps"

Sebastien Michelin[1,*] and Eric Lauga[2,†]

[1] *LadHyX – Département de Mécanique, Ecole Polytechnique – CNRS, 91128 Palaiseau, France.*
[2] *Department of Applied Mathematics and Theoretical Physics, University of Cambridge, CB3 0WA, United Kingdom.*

I. FLOW RATE FOR PHORETIC PUMPS WITH $P = 3$ PATCHES

The general expression for the pumping rate was obtained in Eq. (8) of the main text:

$$Q/L = \sum_{n=1}^{\infty} \frac{h\,\tanh(2\pi nh)}{\pi^2 n^2} \sum_{p<q} \alpha_{pq} \sin(\pi n l_p) \sin(\pi n l_q) \sin\left(\pi n \left[l_p + \sum_{j=p+1}^{q-1} l_j + l_q\right]\right). \tag{1}$$

For $P = 3$, this becomes

$$Q/L = \sum_{n=1}^{\infty} \frac{h\,\tanh(2\pi nh)}{\pi^2 n^2} \sum_{p<q} \Big\{\alpha_{12} \sin(\pi n l_1) \sin(\pi n l_2) \sin(\pi n [l_1 + l_2]) + \alpha_{23} \sin(\pi n l_2) \sin(\pi n l_3) \sin(\pi n [l_2 + l_3]) + \alpha_{13} \sin(\pi n l_1) \sin(\pi n l_3) \sin(\pi n [l_1 + 2l_2 + l_3])\Big\}. \tag{2}$$

Using $l_1 + l_2 + l_3 = 1$, we can write

$$\sin(\pi n(l_1 + l_2)) = (-1)^{n+1} \sin(\pi n l_3) \tag{3}$$

$$\sin(\pi n(l_2 + l_3)) = (-1)^{n+1} \sin(\pi n l_1) \tag{4}$$

$$\sin(\pi n(l_1 + 2l_2 + l_3)) = (-1)^{n} \sin(\pi n l_2), \tag{5}$$

so that finally, with $\alpha_{31} = -\alpha_{13}$,

$$Q/L = (\alpha_{12} + \alpha_{23} + \alpha_{31}) \sum_{n=1}^{\infty} \frac{(-1)^{n+1} h\,\tanh(2\pi nh)}{\pi^2 n^2} \sin(\pi n l_1) \sin(\pi n l_2) \sin(\pi n l_3). \tag{6}$$

II. FLOW RATE FOR PHORETIC PUMPS WITH $P = 4$ PATCHES

Equation (1) can be rewritten now for $P = 4$ with $l_1 + l_2 + l_3 + l_4 = 1$ as

$$Q/L = \sum_{n=1}^{\infty} \frac{(-1)^{n+1} h\,\tanh(2\pi nh)}{\pi^2 n^2} \Big[\alpha_{12} \sin(\pi n l_1) \sin(\pi n l_2) \sin(\pi n[l_3 + l_4]) + \alpha_{23} \sin(\pi n l_2) \sin(\pi n l_3) \sin(\pi n[l_1 + l_4]) + \alpha_{34} \sin(\pi n l_3) \sin(\pi n l_4) \sin(\pi n[l_1 + l_2]) + \alpha_{41} \sin(\pi n l_4) \sin(\pi n l_1) \sin(\pi n[l_2 + l_3]) + \alpha_{13} \sin(\pi n l_1) \sin(\pi n l_3) \sin(\pi n[l_4 - l_2]) + \alpha_{24} \sin(\pi n l_2) \sin(\pi n l_4) \sin(\pi n[l_1 - l_3])\Big], \tag{7}$$

or equivalently

$$Q/L = \sum_{n=1}^{\infty} \frac{(-1)^{n+1} h\,\tanh(2\pi nh)}{\pi^2 n^2} \sum_{j=1}^{4} \mathcal{F}_j \cos(\pi n l_j) \prod_{k\neq j} \sin(\pi n l_k). \tag{8}$$

---

*Electronic address: sebastien.michelin@ladhyx.polytechnique.fr
†Electronic address: e.lauga@damtp.cam.ac.uk

Here, $\mathcal{F}_1 = \alpha_{23} + \alpha_{34} + \alpha_{42}$, is the characteristic pumping rate of the 3-pump obtained with $l_1 = 0$ and the other $\mathcal{F}_j$ are obtained by circular permutation. One note immediately that

$$\mathcal{F}_1 + \mathcal{F}_3 = \alpha_{12} + \alpha_{23} + \alpha_{34} + \alpha_{41} = \mathcal{F}_2 + \mathcal{F}_4, \tag{9}$$

so that the four quantities $\mathcal{F}_i$ are not independent and, as expected, the pumping rate depends only on three independent chemical parameters.

III. OPTIMAL PHORETIC PUMPS FOR $P = 4$

For a given set of chemical properties (i.e. set values of $\mathcal{F}_j$), the pumping rate is the superposition of four different contributions associated with each $\mathcal{F}_j$. In Eq. (8), the contribution proportional to $\mathcal{F}_j$ can be interpreted as a modulation of the pumping rate of the 3-patch pump obtained for $l_j = 0$, resulting from the introduction of a fourth patch. As a result, this contribution is maximum in magnitude for $l_j = 0$ and $l_{k\neq j} = 1/3$ (the center one of the faces of the tetrahedron in Figure 4 of the main text), decreases monotonously away from this maximum and vanishes on all three other faces (where one of the other l_k vanishes).

For given $\mathcal{F}_j$, two cases can therefore be identified for the geometric variations of Q with $(l_j)_j$: the optimal pumping rate $|Q|$ is reached either within $\mathcal{I}_4 = \left\{0 \leq l_i \leq 1,\, \sum_{i=1}^4 l_i = 1\right\}$ or on its boundaries. In the latter case, the optimal pump is degenerated and includes only three different patches: in that case, the optimal is necessary reached for three patches of equal lengths. In the former case, the optimal pump consists of four distinct patches.

In order to distinguish between these two situations, a necessary condition for the 3-patch pump with $l_1 = 0$ and $l_{j\neq 1} = 1/3$ to be a local optimal requires the pumping rate Q_m of that configuration and the derivative of Q with respect to l_1 to have opposite signs (increasing l_1 then reduces $|Q|$). The latter quantity, noted here $\delta Q/\delta l_1$, corresponds to the gradient of Q with respect to $(l_i)_{1\leq i\leq 4}$ projected onto the constraint $\sum l_i = 1$.

These two quantities are obtained (for $l_1 = 0$ and $l_2 = l_3 = l_4 = 1/3$) as

$$Q_m = L\mathcal{F}_1\mathcal{K}_1(h), \quad \left(\frac{\delta Q}{\delta l_1}\right) = L\mathcal{F}_3\tilde{\mathcal{K}}_1(h) \tag{10}$$

with

$$\mathcal{K}_1(h) = \sum_{n=1}^{\infty} \frac{h(-1)^{n+1}\tanh(2\pi n h)}{\pi^2 n^2} \sin^3\left(\frac{\pi n}{3}\right), \tag{11}$$

$$\tilde{\mathcal{K}}_1(h) = \sum_{n=1}^{\infty} \frac{h\,\tanh(2\pi n h)}{\pi n} \sin^2\left(\frac{\pi n}{3}\right). \tag{12}$$

The functions $\mathcal{K}_1(h)$ and $\tilde{\mathcal{K}}_1(h)$ in Eqs. (11)–(12) being strictly positive for all h, the 3-patch pump with $l_1 = 0$ is therefore a local optimum if and only if $\mathcal{F}_1\mathcal{F}_3 < 0$ (it should be noted that the 3-patch pump with $l_3 = 0$ is then also a local optimum). As a result, a classification of the optimal 4-patch pump is obtained for given chemical properties (i.e. given $\mathcal{F}_i$):

1. If $\mathcal{F}_1\mathcal{F}_3 < 0$ (resp. $\mathcal{F}_2\mathcal{F}_4 < 0$), the optimal pump has only three patches, with $l_1 = 0$ or $l_3 = 0$ (resp. $l_2 = 0$ or $l_4 = 0$).

2. If $\mathcal{F}_1\mathcal{F}_3 > 0$ and $\mathcal{F}_2\mathcal{F}_4 > 0$, then the optimal 4-patch pump is not degenerated and features four patches of non-zero lengths. One could further show using a similar method that the pump with four patches of equal lengths is only optimal when the four constants $\mathcal{F}_j$ are all equal.

This distinction exemplifies again the loss of universality of the optimal phoretic pump when $P > 3$.
